# Role of Mechanotransduction in Cancer: A Complex Problem Involving Gene Mutations and Altered Levels of Connection Components

**DOI:** 10.3390/biom16081147

**Published:** 2026-08-07

**Authors:** Frederick H. Silver

**Affiliations:** Department of Pathology and Laboratory Medicine, Robert Wood Johnson Medical School, Rutgers, The State University of New Jersey, Piscataway, NJ 08854, USA; silverfr@rutgers.edu

**Keywords:** collagen, integrins, Talin, kindlin, Paxillin, FAK, mechanotransduction pathways, cell cytoskeleton, nucleoskeleton, intermediate filaments, nuclear lamins, cadherins, caterins, cell nucleus

## Abstract

Background: External and internal forces and tissue energy influence the structure and function of mammalian tissues during life in a gravitational field. Changing force (stress) and energy equilibria provide a dynamic means to regulate cell and tissue growth during development and maturation. However, genetic mutations and changes in expression of macromolecules involved in cell and extracellular matrix (ECM) equilibria lead to tumor formation. Methods: A model is presented illustrating connections between ECM, cell membranes, cell cyto- and nucleoskeletons, cell nucleus, and cell–cell junctions that promote energy storage, transmission, and dissipation. The effects of mutations involving changes in P53 and Coll 11A1 genes and changes in expression of collagens and collagen receptors, integrins, ILK, FAK, Talin, Paxillin, Kindlins, c-SRC, Actin, myosin light chain, Filamin A, E-cadherin, and beta catenin that have been reported to occur in cancerous lesions are examined. Results: When mutations or altered component expressions occur, mechanotransduction pathways are activated that lead to modified epithelial–mesenchymal (EMT) and endothelial–mesenchymal (ENT) transitions resulting in new cell division and deposition of ECM. Conclusions: It is hypothesized that changes in genes and expression of proteins in the connections between ECM and bound cells alter energy storage and dissipation. This leads to local stress concentrations that alter force and energy dynamic equilibria required to maintain homeostasis. Excess energy associated with broken connections within cells is dissipated through changes in myosin structure and function.

## 1. Introduction

**Figure 1:**
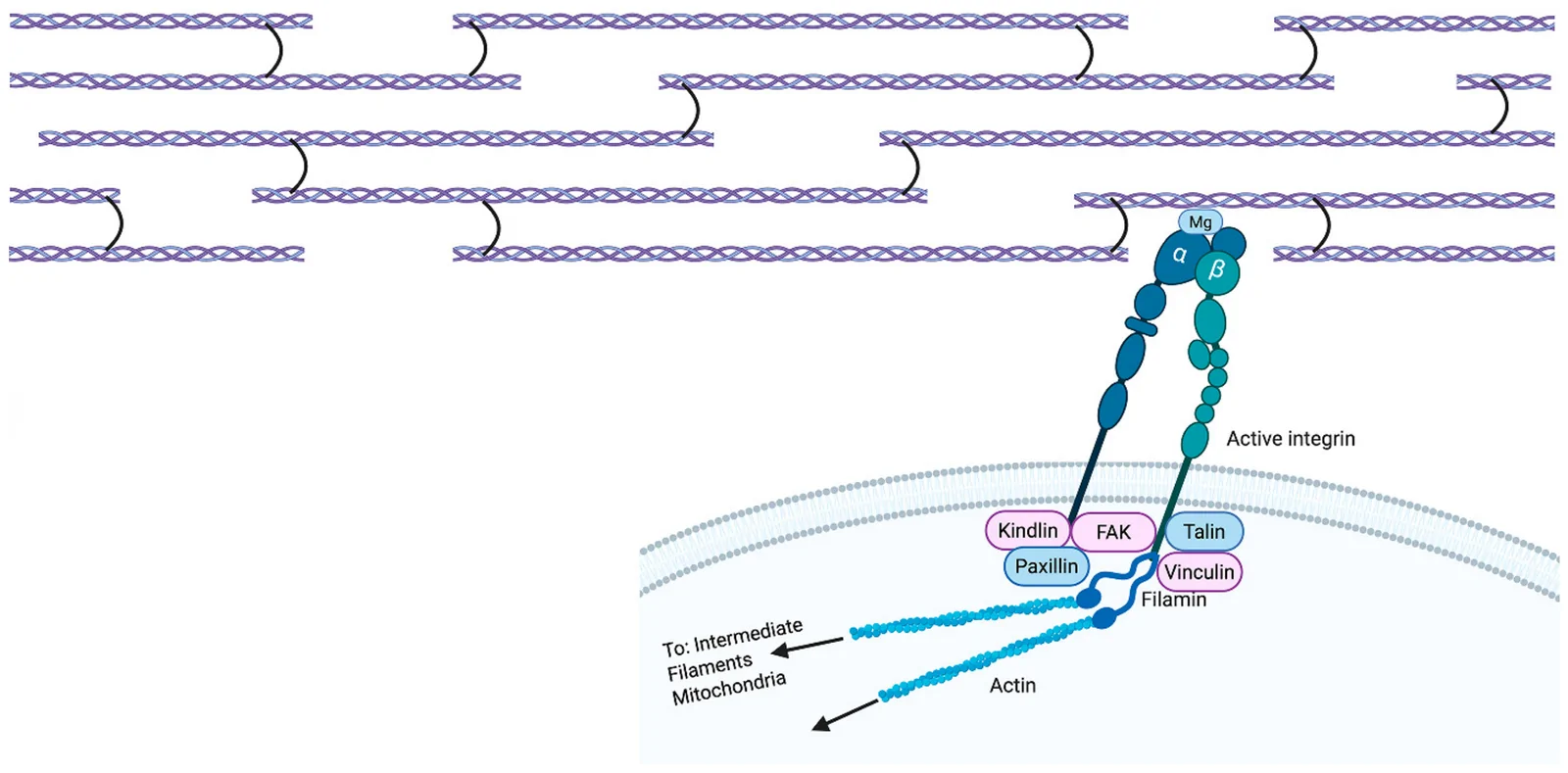
Diagram depicting the mechanical connections between extracellular matrix collagen fibrils and a cell. Note arrows depict connections to other cell components. Collagen fibril (top), alpha2 beta1 integrin components and kindlin, Talin, Vinculin, FAK and Paxillin are part of the infinite connections linking ECM, integrins, Actin microfilaments attached to the cyto- and nucleoskeleton and the cell nucleus. Note that a magnesium ion putatively links integrin alpha2 beta1 to a collagen microfibril in the collagen hole region. Gene mutations, amplification, or increased levels of macromolecular components are associated with tumor progression. It is hypothesized that breakage or alterations of these linkages lead to stress and energy concentration in tissues, leading to epithelial–mesenchymal and endothelial–mesenchymal transitions that influence local cells through altered mechanotransduction pathway activation. Created with BioRender.com.

**Figure 2:**
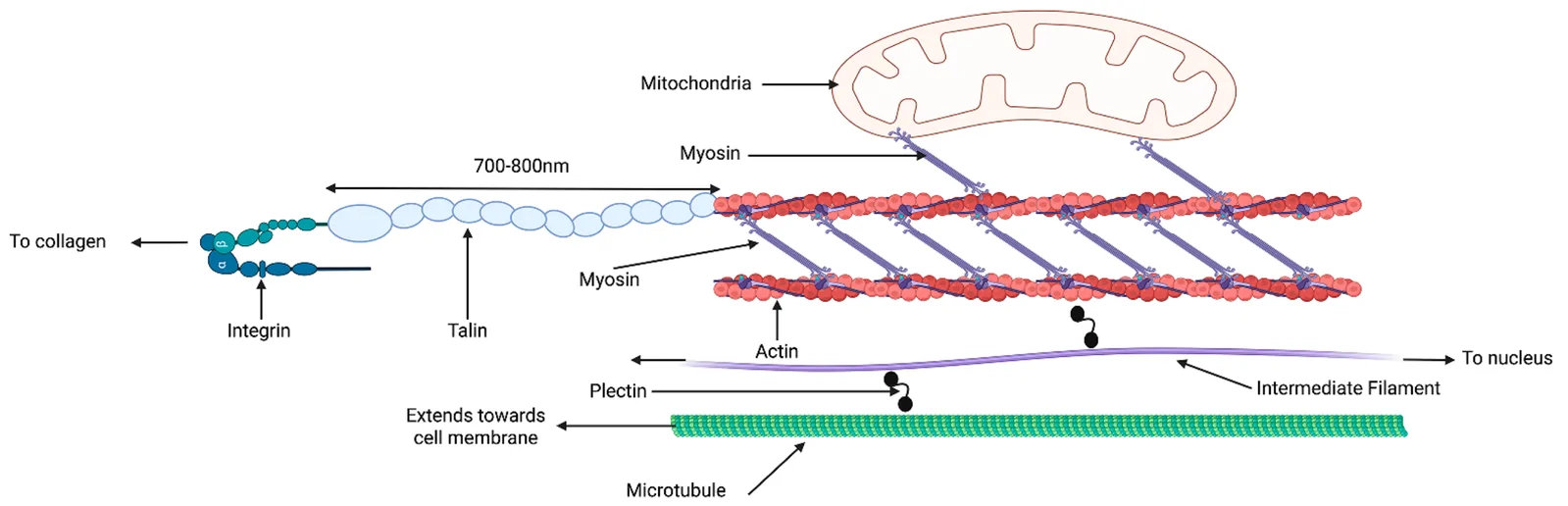
Diagram of the connections between integrins, Talin, myosin, Actin microfilaments, microtubules, and intermediate filaments that connect to nuclear lamins and the cell nucleus. Increased levels or dysregulation of these proteins, gene copy alterations, structural changes in microfilaments, or myosin light chain alterations are observed in cancerous tissues. Breakage or modification of any of these linkages can lead to stress and energy concentrations that modify mechanotransduction pathways and promote cancerous tissue changes. Created with BioRender.com.

**Figure 3:**
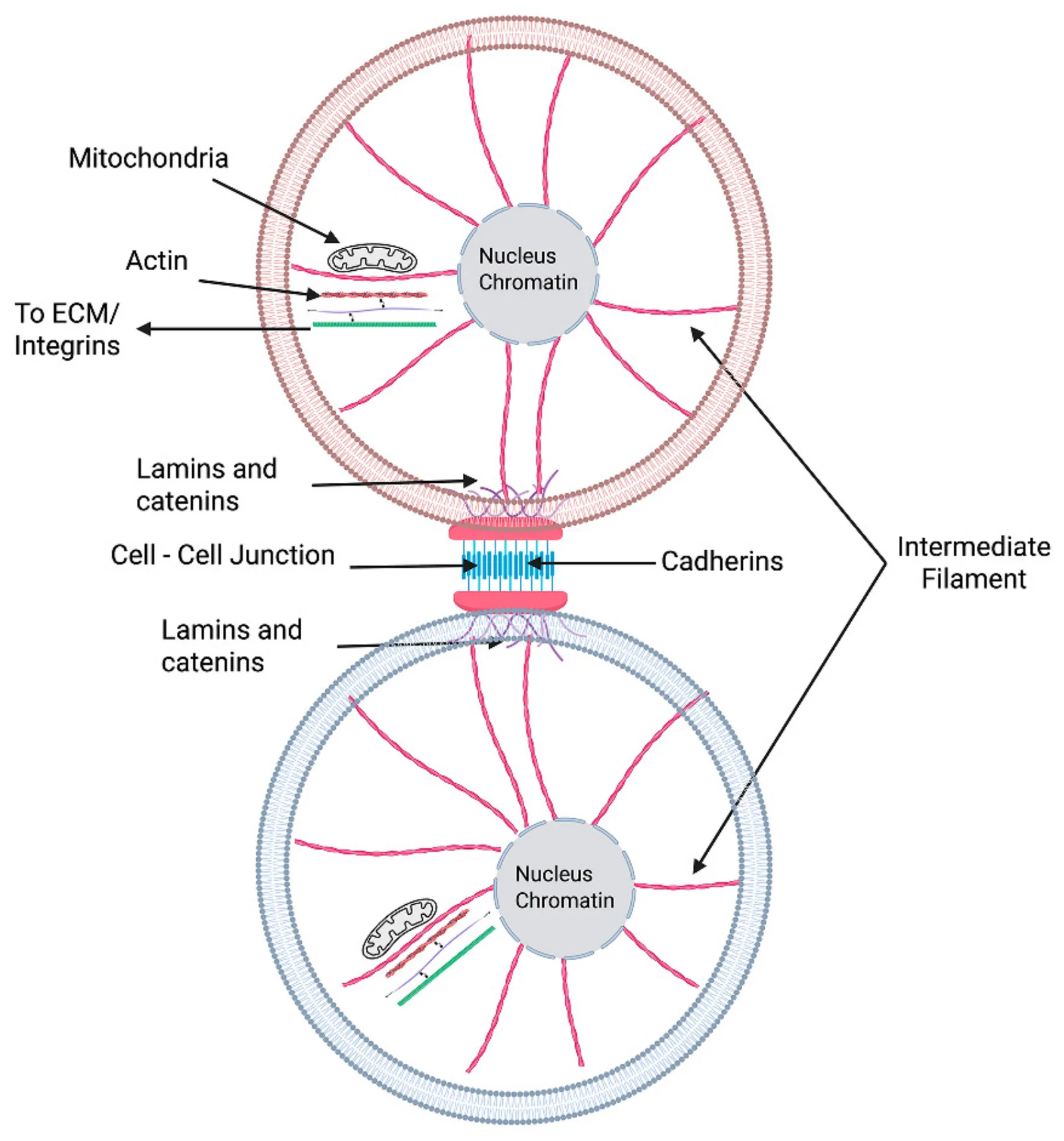
Illustration of the components providing cell–cell connections transferring forces and energy from the cell cytoskeleton to the cell nucleus and between neighboring cells. Connections between collagen fibrils in the extracellular matrix and cell membrane integrins occur through a magnesium found in the hole region of collagen fibrils. Integrins and associated molecules are connected to Actin, myosin, microtubules, mitochondria, and intermediate filaments. Intermediate filaments in turn are connected to nuclear lamins, cadherins, and catenins which are associated with the genetic material in the cell and neighboring cells. In this manner, external forces and energy are transmitted through the tissue into the attached cells and to the nucleus and back out to the extracellular matrix. Alterations in genes, such as p53 or COLL11A, and changes in expression of intermediate filaments, nuclear lamins, cadherins, and catenins prevent force and energy transmission from the cytoskeleton to the cell nucleus and to neighboring cells can cause changes in force and energy equilibria, driving stress concentrations that lead to cancerous changes in the tissue. These changes are associated with tumor formation and metastasis of cancerous lesions and lead to mechanical discontinuity in the ECM–nucleus–ECM force and energy balances. Created with BioRender.com.

Mechanotransduction involves the conversion of mechanical forces and mechanical energy (forces applied through a displacement) into cellular and tissue alterations. These forces and energy come from living in a gravitational field and the resulting retractive forces exerted by cellular and tissue tension. Forces and energy applied to cells and tissues are transferred into the cytoskeleton and then through the nucleoskeleton to the nucleus and back out to the ECM. This creates dynamic equilibria involving forces and energy that ultimately influence tissue structure and function [1]. Mechanotransduction regulates a number of tissue responses to mechanical loading such as changes in skin structure [2], dynamic bone remodeling [3], maintenance of cartilage structure [4], peripheral nerve regeneration [5], regulation of vascular response to exercise [6] and mechanical responses of the eye associated with application of internal and external forces that occur in eye diseases [7]. A recently proposed multiscale mechanical model describes the connections that store, transmit, and dissipate forces and energy in cells and tissues [8]. The connections include collagen fibrils, integrin components, cell cyto- and nucleoskeletal components, Actin microfibrils, microtubules, intermediate filaments, nuclear lamina, cell junctions, and mitochondria [8] (see Figure 1, Figure 2 and Figure 3). Through these connections mechanical information is transmitted into and out of the cell nucleus that impacts activation of several mechanotransduction pathways that control cell and tissue shape and function. Overexpression or under expression of any of the hundreds of molecules involved in these connections and mechanotransduction pathways can lead to disease or structural changes in cells and tissues. These connections allow for forces and energy to be stored, transmitted, and dissipated in cells and tissues that reflect the dynamic state of external and internal loading. For instance, mutations in muscle titin molecules lead to several different diseases, including neuromuscular disorders [9,10]. The purpose of this paper is to discuss some of the genetic mutations and cellular alterations in cells and tissues that involve cell connections. The wide array of molecules that affect cell and tissue mechanical behavior make understanding the pathobiology of cancer formation complex.

Some of the molecules that are associated with the connections between cells and extracellular matrix (ECM) are listed in Table 1. Table 2 summarizes the location of these components and their roles in connecting elements in the ECM and cell. Table 3 relates the components to some of the mechanotransduction pathways that are activated by application of forces and energy.

## 2. Methods

### 2.1. Importance of Energy Storage, Transmission, and Dissipation Between Cells and Tissues

How forces and energy are stored, transmitted, and dissipated by cells and tissues dictates whether a tissue will tear, or if mechanotransduction is activated promoting changes in cell and tissue structure. Force and energy transfer between the ECM and cell and back again involves extracellular matrix components as well as components of the cell as discussed in this section. When these connections are disrupted by either mutations or dysregulation, force and energy balances are putatively disrupted.

One of the physiological functions of extracellular matrix is to dissipate impact loads and prevent mechanical failure from stress concentrations [8]. Fibrosis and stiffening of the tissue around cells and tissues prevent energy transmission and dissipation of applied forces and energy, thereby promoting local accumulation. Loading of cells and tissues containing genetic mutations and modified macromolecular connections can activate mechanotransduction pathways and lead to cellular modifications, fibrosis, and functional failure such as occurring in cancer. Processes such as the epithelial-to-mesenchyme and endothelial-to-mesenchyme transition are involved in changes in the composition and structure of the tumor environment.

Energy transmission in cells and tissues occurs through the covalent and ionic connections between macromolecular structures that form an infinite structural network. Energy storage in tissues occurs via reversible conformational changes in the structural molecules like collagen or through protein synthesis [8]. In contrast, energy dissipation occurs via fluid redistribution within cells and extracellular matrix (ECM), generation of heat, changes in myosin and motor protein structure, or via flow in the cardiovascular system [11]. Forces and energy applied to tissues that do not lead to mechanical failure are: (1) stored reversibly in collagen fibrils and fibers during uncoiling of flexible triple helical sections [8]; (2) transmitted downstream to attached cells and tissues via covalent and ionic connections [8]; and (3) dissipated as heat through fluid flow or through connections to the mitochondria and myosin [12].

Energy transmission occurs through doing work (force applied through a displacement is energy that can be applied to a cell) via mechanical connections formed between macromolecular assemblies both outside and inside the cell. Work and energy applied to cells and ECM components is either transmitted away from the point of application, dissipated, or the tissue will stretch and eventually tear. In most tissues, force and energy transmissions occur between collagen fibrils in the ECM, and integrins and their cofactors, that connect the ECM and the cyto- and nucleoskeletons. Forces and energy are transferred through intermediate filaments and nuclear lamins to the cell nucleus [8]. If one or more of these connections are modified or broken, then energy transmission and storage either lead to tissue mechanical failure in the form of a stress fracture (bone), or to an epithelial–mesenchymal transition (EMT) and an endothelial–mesenchymal transition (ENT) that can lead to tumor formation and growth. This occurs due to the accumulation of stress at a point where the connections are broken or modified in the cell. Overexpression, under expression, and genetic mutations of molecules involved in the connections between these elements can lead to changes in activation of mechanotransduction pathways [13]. Altered activation of mechanotransduction pathways ultimately leads to new homeostatic equilibria between cells and ECM that modify cell and tissue composition and structure [1].

### 2.2. Changes in Mechanotransduction in Cancer

Changes in many of the proteins involved in the connections between the ECM and the cell nucleus are reported in cancer. This suggests that these connections, when broken or modified can enhance local stress concentrations perturbing the current operating force and energy equilibria to prevent tissue fracture and tearing. Excess local energy activates mechanotransduction pathways inside cells and tissues leading to epithelial–mesenchymal (EMT) and endothelial–mesenchymal transitions (ENT). This leads to synthesis of new cells and ECM that alters tissue structure and function [14]. In this paper, the relationship between changes in cell and tissue macromolecular structure and function will be postulated in relationship to their effects on mechanotransduction and energy utilization within the cell. These transitions have a major influence on what happens in wound healing and in cancer [14]. Below, the changes in the components involved in these connections and their relationship to formation of cancerous tumors are discussed. The changes observed in cancerous tissues include mutations, overexpression, and under expression of components involved in the connections between ECM and cells. Both genetic mutations and changes in protein expression and structure lead to alteration in mechanotransduction pathways in cancer.

### 2.3. Collagen Mutations and Cancer

Mutations in collagen genes and abnormal collagen deposition play a defined role in progression of cancer. P53 is a tumor suppressor gene that is commonly mutated in cancer [15]. It stops the formation of tumors; mutations in p53 occur in more than 50% of human primary tumors [16]. Mutant p53 stimulates cancer cell invasion through enhanced Rho/Rock-dependent cell-mediated ECM reorganization [17] (see Table 3). There is some indication that the Rho/Rock pathway may be linked to ERK signaling that is part of the MAPK pathway [18]. These mutations lead to increased secretion of fibronectin and collagen I, III and IV [19]. Mutant p53 may support tumor-promoting functions of TGF-beta allowing cancer cells to proliferate [20]. TGF-beta activates Smad signaling which leads to Smad-mediated transcriptional regulation [21]. Crosstalk between TGF-beta/SMAD and MAPK pathways has been reported [22]. TGF-Beta/SMAD 3 signaling promotes collagen synthesis in the pulmonary artery [23]. Crosstalk between the ERK/MAPK-SMAD signaling pathway enhances TGF-Beta responses in certain cell types [24]. TGF-beta activation has been recognized as the source of collagen deposition through EMT in wound healing and in cancer.

In addition to p53 mutations, COL11A1 mutations occur in most squamous cell carcinomas (cSCCs) of the skin [25]. These mutations are found in a triple helical region known to disrupt normal collagen fibrillar structure [26]. COL11A1 encodes the Type XI collagen α1 chain, known to regulate the structure of fibrillar collagen [27]. COL11A1 overexpression has been associated with reduced cancer survival in multiple studies [28,29,30,31]. COL11A1 overexpression is associated with decreased cancer survival of head and neck, breast, ovarian, colon, and lung lesions, suggesting a role for COL11A1 in a wide spectrum of cancers [28,29,30,31]. Collagen Type X1 is normally found in cartilage but mutations in this gene result in deposition of abnormal Type I collagen in skin cancers. Abnormal collagen expression in the tumor may disrupt energy storage, transmission, and dissipation in cells, altering force and energy balances in the affected tissue. There are other genetic mutations besides p53 and COL11A1 in cancer that will be included in future models.

Besides integrin receptors for collagen, discoidin receptors are also found on the cell surface. Discoidin domain receptor 1 (DDR1) is a tyrosine kinase receptor that is specifically activated by collagen [32]. DDRs can activate signal transduction pathways, such as the MAPK pathways (See Table 3), that regulate cell-collagen interactions involved in pathological processes [33]. DDR1 binding to collagen which occurs through the discoidin domains is integrin-independent. Following activation by collagen, DDR1 triggers the activation of downstream signaling pathways [34], inducing the expression of pro-inflammatory mediators as well as matrix-degrading enzymes. Overexpression of DDR1 has been reported in several cancers [35], including breast and lung cancers [36,37,38,39].

The discoidin domain receptor 1 (DDR1) tyrosine kinase is a direct mechanosensor and is involved in flow-induced endothelial cell activation of DDR1. Shear force likely causes conformational changes in DDR1 that facilitate force-induced receptor aggregation [40]. DDR1 expression is associated with fibrosis in heart, kidney, liver, lung and perivascular tissues. Dysregulated collagen remodeling is associated with mechanical signaling functions of DDR1 in processing of fibrillar collagen that leads to tissue fibrosis [41]. Table 4 [39,40,41,42,43,44,45,46,47,48,49,50,51,52,53,54,55,56,57,58,59,60,61,62,63,64,65,66,67,68,69,70,71,72,73,74,75,76,77,78,79,80,81,82,83,84,85,86,87,88,89,90,91,92,93] summarizes some of the changes in genes and proteins found in cancerous tissue, and Table 5 provides information on the pathways affected by elements in Table 4.

### 2.4. Molecules Involved in Mechanotransduction in Cancer

#### 2.4.1. Integrins

Integrins and discoidin domain receptors (DDRs) are the two major and distinct transmembrane receptor families, that serve as primary sensors for collagens. Both can activate shared downstream pathways and their functional interplay remains complex with the potential to fine-tune cellular responses to ECM cues [42]. Deregulation of signaling and its various consequences are important to cancer [43]. Abnormal expression of integrins is part of a multistep process of cancer progression, immunosuppression, and resistance to treatment [43]. Integrins regulate and maintain tumor characteristics by activating various signaling pathways including TGF-beta, FAK-AKT, or ERK [44,45,46,47] (see Table 3 and Table 5). In metastatic colorectal cancer, the integrin α2β1 signal interacts with Cadherin-17, recruiting Talin, thereby facilitating proliferation and metastasis of metastatic colorectal cancer [48]. ITGB1 (integrin) gene codes genetic instructions for making beta-1 integrin protein. p38 MAPK kinase phosphorylation is regulated by integrin α2β1 promoting both cell proliferation and inhibition in the process of regulating tumor proliferation [43] (see Table 3). Altered integrin expression patterns have been linked to many types of cancer [41].

For example, integrins αvβ3, αvβ6, and α5β1 are usually expressed in most normal epithelia at low or undetectable levels but can be highly upregulated in multiple tumors [49]. The overexpression of the integrins αvβ3, αvβ5, αvβ6, α5β1, α6β4, and α4β1 promotes cancer progression in various cancer types [49]. Integrin-mediated signaling pathways, including Ras- and Rho-GTPase, TGFβ, Hippo, Wnt, Notch, and sonic hedgehog are involved in various stages of tumorigenesis (see Table 3) [50]. Therefore, the complex regulatory mechanisms and molecular specificities of integrins involve understanding the relationship between integrin modifications and several mechanotransduction pathways [50].

#### 2.4.2. FAK

FAK activation promotes tumor growth, invasion, metastasis, and angiogenesis via both MAPK-dependent and MAPK-independent pathways [51]. It is activated by integrins and growth factors and is required for growth factor activation of the MAPK pathway [52]. FAK can influence extracellular matrix production and exosome secretion through cancer-associated fibroblasts; thus, it has an important role in tumor microenvironment regulation [53].

#### 2.4.3. Talin

Talin-1 expression levels are correlated with invasion and lower survival rates in prostate cancer, colon cancer, nasopharyngeal carcinoma, and oral squamous cell carcinoma (SCC) [54,55,56]. Talin-1 knockout in prostate cancer and colorectal cancer cell lines reduces cell migration and proliferation [57,58]. Aggressive tumor behavior may be associated with Talin1 protein and with more aggressive tumor behavior and advanced disease in patients with skin cancers [59]. Point mutations in Talin-1 can affect cell behavior and may contribute to cancer progression [60]. High expression of Talin-1 is associated with tumor progression and recurrence in melanoma skin cancer patients [60].

#### 2.4.4. Vinculin

A Vinculin–Actin complex is involved in ECM stiffness-dependent regulation of Vinculin behavior [61]. Vinculin expression is significantly lower in cancer tissues than in paracancer tissues. A total of 5-year overall survival rate is significantly higher for Vinculin-positive patients compared to Vinculin-negative patients [63]. Vinculin-Actin interactions participate in focal adhesion-mediated mechanotransduction [64]. An Actin-binding-deficient mutation disrupts the extracellular matrix (ECM) stiffness-dependent regulation of CSB (cytoskeleton stabilization buffer) resistance and the stable localization of Vinculin [64].

#### 2.4.5. Paxillin

Somatic mutations of Paxillin family members in cancer are generally considered rare; however, they may play a role in some cancers [65]. The Paxillin family of proteins are involved in cancer cell morphology, migration, and invasion. Their roles in crosstalk with the MTs, MFs, and IFs in the cytoskeleton help modulate cell polarity, trafficking, and ECM remodeling. Paxillin regulates MT acetylation by binding to and inhibiting it [66]. Depletion of Paxillin results in a significant decrease in MT acetylation but does not affect overall tubulin expression or distribution [66]; however, increased MT acetylation is associated with aggressive cancers.

#### 2.4.6. Kindlins

Kindlins are reported altered in more than 10,000 patients with 33 cancer types in advanced tumor stages and in metastatic cancers [67]. Co-alteration between kindlins and pathways in various cancers adversely affects survival outcomes [68] and dysregulates integrin-signaling promoting tumor progression [68].

#### 2.4.7. c-Src

SRC controls the behavior of transformed cells and contributes to tumor progression and metastasis. c-Src has been implicated in several human cancers; for example, elevated c-Src activity has been found in human sarcoma tissues, head and neck cancer, lung cancer, breast cancer, and various other carcinomas [69]. c-Src upon activation activates multiple downstream signaling pathways, including PI3K-AKT, Ras-MAPK, JAK-STAT3, and the FAK/Paxillin pathways (see Table 3), which are important for cell proliferation, survival, migration, invasion, metastasis, and drug resistance [69]. Overexpression and overactivation of SRC have been observed in numerous cancer types [70]. The increased SRC activity found in cancer cells can be caused by multiple factors, including an enhanced expression of SRC activators [70].

#### 2.4.8. Actin

Actin expression changes have been reported in cancer. Non-muscle Actin is increased in liver, melanoma, renal, gastric, pancreatic, esophageal, lung, breast, prostate and ovarian cancers and in leukemia and lymphoma [71]. Gene alterations include mutations, gene fusions, and copy number alterations (deletions and amplifications). Their occurrence is observed in lymphoid cancers, nonmelanoma skin cancer, and Actin gene copy number alterations in breast, prostate and liver cancers [71].

#### 2.4.9. Myosin

Non-muscle myosins (NMIIs) and their regulators have been detected in cancer cells [72]. Myosin light chains are implicated in causing cancer in different organs and tissues. Changes in the Myosin light chain 1 (MYL1) promote tumor metastasis in head and neck squamous cell carcinoma and are an unfavorable marker of the disease [73]. Connections between myosin and Actin are an important part of the links between components of the cytoskeleton, as diagramed in Figure 2.

#### 2.4.10. Filamin

The low expression of Filamin A is considered a potential driver gene of metastasis, and its low expression enhances the 5-year relapse survival rate by 15% [74]. Low levels of Filamin An expression in cancer cells are more often associated with metastasis-free survival than those with normal levels of Filamin-A [75]. Filamin A is a suggested prognostic marker for cancer metastasis, but inhibition of Filamin A in cancer cells may reduce metastasis [75]. Mutations in the Filamin A gene, which encodes the protein, are linked to various neurodevelopmental disorders, including periventricular nodular heterotopia, characterized by the abnormal accumulation of neurons that fail to migrate correctly during brain development [76]. Filamin A is highly expressed in triple negative breast cancer [78]. It shows conflicting roles considering its ability to bind, regulate, and work with at least 90 different proteins and the differential roles that it can have depending on the subcellular localization [77]. Filamin A is an important molecule that connects integrin alpha2 beta 1 to Actin microfilaments (see Figure 1).

#### 2.4.11. Plectins

Dysregulation of plectins occurs in cancer. Both the expression levels and subcellular localization of plectins are frequently dysregulated in cancer [78]. Their dislocalization has been shown to confer unexpected functions in cancer progression, including the promotion of tumor growth and metastasis, as well as the maintenance of cancer stem cells stemness [79]. The plectin gene encodes a cytoskeletal linking protein known for its interaction with three critical components of the cellular cytoskeleton: intermediate filaments, microtubules, and Actin filaments [78] (see Figure 2). In recent years, studies have reported that plectins are closely related to tumorigenesis and development, exhibiting both tumor-suppressive and tumor-promoting functions [79].

#### 2.4.12. Intermediate Filaments and Nuclear Lamins

There are six types of intermediate filaments (IFs) (see Figure 3). Types I–IV can be found in the cytoplasm of various cells. Type V intermediate filaments consist of lamins located in the nucleus, also known as nuclear lamins [80,81]. Various biomarkers found in cancer tissues underscore the role of intermediate filament components, specifically vimentin, nestin, and keratin [81]. In cancer, IF proteins serve as diagnostic markers, as tumor cells partially retain their original signature expression of IF proteins. However, there are also characteristic alterations in IF gene expression and protein regulation [82]. One study revealed that intermediate filament family orphan 1 (IFFO1) is downregulated in lung cancer patients and is correlated with a poorer prognosis [82]. The depletion of IFFO1 led to enhanced tumor proliferation both in vitro and in vivo [82]. IFF0 is involved in DNA double-stranded break attachment.

#### 2.4.13. Mitochondrial Elements Involved in Cancer

Warburg observed, 70 years ago, that tumors produce excess lactate in the presence of oxygen and undergo glycolysis [83]. Although mutations in mitochondrial genes are common in cancer cells, they do not inactivate mitochondrial energy metabolism but rather alter the mitochondrial bioenergetic and biosynthetic state [83]. Cancer cells often reprogram their metabolic pathways to provide energetic and biosynthetic flexibility. Metabolic reprogramming is considered as one of the major hallmarks of cancer [84] and is perhaps a mechanism by which excess energy associated with stress concentrations causes structural changes in myosin and other motor proteins that promote cell movement. Dysregulated mitochondrial fission and fusion promote tumor progression. Altered mitochondrial fission and fusion change tumor cell growth, metabolism, motility, and invasion indirectly impacting tumor progression to metastasis [84].

#### 2.4.14. Microtubule Changes in Cancer

Altered expression of tubulin isotypes is a hallmark in a range of cancers. Analysis of many cancers shows a high expression of several β-tubulin isotypes correlating with aggressive clinical behavior, chemotherapy drug resistance, and poor patient outcome [85]. An increase in βI tubulin expression is observed in several cancers [86,87].

#### 2.4.15. E-Cadherin

E-cadherin is crucial for the adhesion between cells and the formation of epithelial tissues [88]. E-cadherin is involved in cell signaling via its intricate interactions with various signaling pathways such as the Wnt/β-catenin pathway, receptor tyrosine kinases (RTKs), Janus kinase/signal transducers and activators of transcription (JAK/STAT) pathway, transforming growth factor (TGF-β), and nuclear factor kappa-light chain-enhancer of activated B cells (NF-Κb) signaling pathway [89]. Loss of E-cadherin is observed in almost all types of cancer limiting cell–cell adhesion, leading toward malignancy. Downregulation of E-cadherin expression has been linked to poor overall survival and prognosis of malignancies [88]. During EMT, the loss of E-cadherin results in the translocation of β-catenin to the nucleus, which activates signaling pathways that lead to cell proliferation [88,89,90].

#### 2.4.16. Beta-Catenin

Beta-catenin is involved in the WNT/beta-catenin signaling pathway. Accumulation of beta-catenin in the nucleus occurs after promotion of oncogenes leading to tumor progression [91]. It acts both as a transcriptional co-regulator and an adaptor protein for intracellular adhesion. β-catenin stabilization is critical for tumorigenesis, which is usually induced by aberrant WNT activation and somatic gene mutations of β-catenin or destruction complex components [91]. With inactivation of the β-catenin-destruction complex, β-catenin accumulates in the cytoplasm, and eventually translocates into the nucleus [92].

#### 2.4.17. Integrin Linked Kinase (ILK)

ILK is a serine/threonine protein kinase that interacts with integrin β1 and the β3 cytoplasmic domains and phosphorylates integrin β1. ILK has multiple functions in cells, such as cell-extracellular matrix interactions, cell cycle, apoptosis, cell proliferation and cell motility. Upregulation of ILK is frequently observed in cancerous tissues compared to corresponding normal tissues [93]. ILK overexpression induces tumorigenic transformation of epithelial cells in vitro and in vivo and is accompanied by upregulation of fibronectin matrix assembly and downregulation of E-cadherin expression [91]. The role of ILK in cancers includes cell proliferation, cell survival, angiogenesis, metastasis and drug resistance [94].

### 2.5. Pathways Involved in Mechanotransduction

There are several pathways involved in mechanotransduction that are either individually stimulated or co-stimulated during cancer. Table 5 Lists some of the pathways that are stimulated in cancer.

**Table 5:** Mechanotransduction Pathways Stimulated in Cancerous Tissues [1].

Pathway	Role
MAPK	Three pathways that are activated during mechanical loading
ERK	Activated by direct growth factor receptor activation
JNK & p38	Activated by stress driving inflammation
ERK 5	Responds to shear stress supports tumor cell survival
Hippo	Phosphorylates YAP/TAZ, unphosphorylated YAP/TAZ enter the nucleus turning on target genes that regulate cell survival
PAM (p13/AKT)	Mutations and overactivation modify tumor mass
Rho, RAS, Rock	Promote cell migration in cancer
Sonic Hedgehog	Involved in cell–cell communication; activation drives tumor growth
WNT/beta-catenin	Excessive activation causes accumulation of beta catenin in nucleus driving expression of oncogenes
JAK-STAT	Overexpression promotes tumor cell survival
FAK/Paxillin	Overexpression in cancer protects cancer cell survival
SMAD	Can suppress growth or can promote tumor growth

There are numerous cellular mutations and changes in cellular component expression that can lead to changes in force and energy balances in cancerous tissues. These changes appear to be consistent with the observations of changes in the amount and types of the cellular and matrix components in skin cancers. Recent experimental studies conducted in vivo indicate that increased cellular stiffness, and increased collagen and fibrotic tissue stiffness are observed in basal cell carcinoma, squamous cell carcinomas, and melanomas [95,96]. These observations are consistent with altered force and energy balances in tumors. Further work is needed to understand how these changes observed in tumors might cause stress and energy concentrations that alter cell and tissue function.

## 3. Conclusions

The interaction of mechanical loading from both external and internal sources has several effects on cells and tissues. The results of mechanical forces and energy on cells are affected by genetic mutations as well by changes in expression of the proteins that form connections between the ECM and cells, and between cells. Mutations discussed include changes in P53 and Coll 11A1 genes while changes in expression of collagens and collagen receptors, integrins, ILK, FAK, Talin, Paxillin, Kindlins, c-Src, Actin, myosin light chain, Filamin A, E-cadherin, and beta catenin have been reported to occur in cancerous lesions. This is accompanied by changes in intermediate filament composition and altered mitochondria acetylation that modify transmission and dissipation of energy in cancer cells. Many of these changes activate one or more of the mechanotransduction pathways and either independently or codependently lead to altered structure and function of the tumor environment. It is hypothesized that changes in genes and expression of proteins are involved in the connections between ECM and bound cells and alter energy storage and dissipation. This leads to local stress concentrations that alter force and energy dynamic equilibria required to maintain homeostasis. Activation of mechanotransduction pathways is a mechanism by which applied forces and energy are stored, transmitted, and dissipated in cells under enhanced loading conditions. This prevents mechanical failure of cells and tissues exposed to external and internal loading. Direct connections between ECM, cell membrane, cell cyto- and nucleoskeleton, cell nucleus and cell–cell components promote energy storage, transmission, and dissipation. When mutations or altered component expressions occur, mechanotransduction pathways are activated that lead to modified EMT and ENT, resulting in new cell division and deposition of ECM. It is hypothesized that these changes result in local stress concentrations that partially dissipate energy via altering myosin and other motor protein conformations promoting cell movement. The complexity of the changes associated with both mutations and expression of molecules connected into infinite mechanical networks found in cells and tissues makes understanding the pathobiology of cancer formation very difficult. This is especially true considering that coactivation of one or more mechanotransduction pathways is likely to occur simultaneously in cancerous tissues.

## Figures and Tables

**Table 1:** Elements and Their Roles in the Multiscale Mechanical Model of Cells and Tissues [1].

Element	Mechanical Role
Actin Microfilaments (MF)	Thin Complexes of Actin that Transfer Force and Energy to and from Nucleus
Beta-catenin	Part of WNT/Beta-Catenin Pathway In Maintaining Tissue Homeostasis, Connect to Cadherin Cell Junctions
Cadherins	Hemi-Desmosome Components that Connect IFs in Neighboring Cells
Collagen Fibrils and Fibers	Store and Transmit Forces and Energy to Cytoskeleton and Nucleoskeleton
c-Src	Mechano-Sensitive Integrin Associated Protein
Discoidin Receptors	Connect to Collagen and Activate MAPK Pathways
Filamin	Crosslinks Actin Microfilaments
Focal Adhesions (FAs)	Contain Integrins, Talin, Vinculin, Kindlin, and Paxillan that Bind Actin Molecules to Collagen
Focal Adhesion Kinases (FAK)	Acts as a Mechanical Sensor and Bridge Between Collagen and the Cytoskeleton
Integrins	Provides a Mechanical Link between Collagen and the Cytoskeleton
Integrin-Linked Kinase (ILK)	Mechano-Sensitive Integrin Associated Protein
Intermediate Filaments (IF)	Cytoplasmic Connection Between Cytoskeleton and Nucleus
Microtubules (MT)	Hollow Tubes Connected to Cell Membrane, Microfilaments, and Nucleus
Mitochondria	Attached to Microfilaments and Microtubules (Produce Energy)
Nuclear Lamina	Connection between IFs and Cell Nucleus-Fibrous Network Lining Inner Nuclear Membrane
Paxillin	Organizes Signaling Molecules in Cytoskeleton
Plectin	Crosslinks IFs
Talin	Mechanosensitive Cytoskeletal Linker Protein
Vinculin	Transmits Forces and Energy Between Cytoskeleton and Collagen

**Table 2:** Cellular Components involved in Mechanotransduction [1].

Structural Component	Effectors of Mechanotranduction
Cell Membrane	Integrins, Discoidin receptors, Ion channels, Growth factor and Hormone receptors, Cadherins, Catenins
Cell Cytoskeleton	Focal adhesions, Actin, Myosin, Intermediate filaments, Microtubules, Mitochondria
Cell Junctions	Tight junctions, Gap junctions, Adherens junctions, Desmosomes,
Nucleoskeleton	Nuclear Lamins, LINC proteins

**Table 3:** Signaling Pathways Involved in Mechanotransduction [1].

Pathway	Role in Mechanotransduction
MAPK	ERK (EGF/ERK ½), cJun, p 38, ERK 5 involved in gene transcription, cell differentiation, cytokine release, and apoptosis
Hippo	Limits cell proliferation, inhibits YAP/TAZ activity, YAP/TAZ activated in cancer, activated by Discoidin receptors
PAM (p13/AKT)	Activated in human cancers, influenced by Growth Factor pathways
Rho, RAS (ROCK)	Encode proteins involved in Cell Signaling and Mutation, cause uncontrolled Growth and Invasion, and Cell death, related to ERK Pathway
Sonic Hedgehog	Activated in development and progression of several cancers
WNT/Beta Catenin	Promotes differentiations of cancer stem cells that are precursors of mature cancer cells
JAK-STAT	Mediates Cellular Responses to Cytokines
FAK/Paxillin	Regulators of N-Cadherin Cell-Cell Adhesion
SMAD (TGF-beta)	Primary intracellular transducer for TGF-beta-binds to TGF-beta, travels to nucleus, transcription factor, activates or represses target genes
Discoidin	Domain Receptors (DDR) tyrosine kinases receptors bind directly to collagen mechanosensors, act like mechanosensors mediating YAP/TAZ activation through the Hippo Pathway

**Table 4:** Changes in Genes and Proteins Associated with Different Cancers.

Element	Cancerous Effect
P53	p53 gene mutations lead to loss of control over cell growth, p53-SMAD complex promotes cell migration [15]
Coll11A1	Gene overexpression by cancer-associated fibroblasts leads to fibrosis [26]
Discoidin	Overexpression of receptors and collagen binding leads to inflammation [32]
Integrins	Interacts with cadherins during overexpression recruiting Talin facilitates cancer cell proliferation
FAK	Gene locus amplification promotes tumor growth by MAPK dependent and independent pathways [52]
Talin	Overexpression increases integrin clustering into focal adhesions [54,55,56]
Vinculin	Underexpression found in cancerous lesions [63]
Paxillin	Overexpression increases microtubule acetylation and cancers [65,66].
Kindlins	Alteration dysregulates integrin signaling and promotes tumors [68,69]
C-SRC	Induces increased pathway expression of P13-AKT, RAS-MAPK, JAK-STAT, Fak-Paxilin [70,71]
Actin	Increased mutant expression in cancers [73]
Myosin	Increased light chain mutations in cancer allowing migration through ECM [74]
Filamin A	Decreased expression enhances survival with cancers [75,76]
Plectin	Has both tumor suppressing and promoting functions [79,80]
IFs	Altered production of IF proteins associated with cancer [81]
Mitochondria	Altered fission and fusion associated with cancer [84,85]
Microtubules	Increased expression of beta tubulin with poor cancer outcomes [86,87,88]
E-cadherin	Loss associated with poor cell–cell adhesion in cancer [89,90,91]
Beta-catenin	Beta-catenin accumulation in nucleus promotes tumors through WNT/beta catenin pathway [91,92]
ILK	Increased expression and interaction with beta1 integrin promotes tumor formation in epithelia [93]

## Data Availability

No new data were created or analyzed in this study. Data sharing is not applicable to this article.
